# Implementation of a Family-Centered Care Model in Psychiatric Hospitals as a Means of Reducing Caregiver Anxiety: A Comparative Study

**DOI:** 10.1007/s10597-025-01551-z

**Published:** 2025-11-03

**Authors:** Ron Shor, Kfir Feffer, Jonathan Guez, Boris Nemetz, Chanoch Miodownik, Vladislava Gurman, Anat Shalev

**Affiliations:** 1 School of Social Work and Social Welfare, The Hebrew University of Jerusalem, 91905 Jerusalem, Israel; 2 Lev-Hasharon Mental Health Center, Tsur-Moshe, Faculty of Medical and Health Sciences, Tel Aviv University, Tel Aviv, Israel; 3 Department of Psychology, Achva Academic College, Beer-Sheva Mental Health Center, Faculty of Health Sciences, Ben Gurion University of the Negev, Beersheba, Israel; 4 Medical School, Psychiatry Division, Beer-Sheva Mental Health Center, Faculty of Health Sciences, Ben Gurion University of the Negev, Beersheba, Israel; 5 Faculty of Social Work, Ashkelon Academic College, Ashkelon, Israel

**Keywords:** Family centered care model, Psychiatric Hospitals, Caregivers, Stress, Anxiety, Mental illness

## Abstract

Caregivers of persons with mental illness experience stressors and anxiety during the psychiatric hospitalization of a family member. The implementation of a family-centered model could reduce these feelings, and therefore, such a model was developed in Israel. To examine whether the model's implementation makes a difference in the stress and anxiety experienced by caregivers, a comparative study was conducted in two psychiatric hospitals. Questionnaires were delivered twice to caregivers in an intervention group (*n* = 93) and a control group (*n* = 75). The anxiety level was lower in the intervention group than in the control group in the second delivery of the questionnaire. In addition, a lower level of anxiety was found in the intervention group in the second delivery of the questionnaire in comparison to the first. The findings, which can be attributed to the model's application, highlight the need for a paradigm shift to a family-centered model in psychiatric hospitals.

## Introduction

Family members of persons with severe mental illness (SMI) who play a caregiving role during the psychiatric hospitalization of a family member often experience stressors and a sense of burden stemming from the hospitalization. This can lead to emotional reactions such as a high level of distress, depression, and anxiety (Ma et al., 2018). The term "caregiver strain" has been used to refer to the negative physical and emotional impact on caregivers resulting from the additional responsibilities of caring for a family member with SMI (Brannan & Heflinger, 2001). Research indicates that caregivers who experience such burden tend to neglect themselves and are at higher risk for developing mental health and medical problems (Johnson et al., 2003; Perlick et al., 2008). However, the stressors experienced by caregivers could differ based on the nature of their relationships with mental health professionals in the psychiatric wards and based on the nature of their involvement in the treatment process during the psychiatric hospitalization. Involvement in the treatment process during the psychiatric hospitalization of a family member with SMI has the potential to reduce stress-provoking situations for caregivers, such as the lack of sufficient knowledge about the mental health situation of their family member who is hospitalized in a closed ward.

The value of supporting caregivers during psychiatric hospitalizations, of minimizing negative outcomes they may experience, and of optimizing the care they provide to the hospitalized family member with SMI has received considerable attention in recent years (Ewertzon et al., 2011; Hsiao et al., 2019; Kokorelias et al., 2019). One model capable of promoting family-member involvement during hospitalization is the Family-Centered Care (FCC) model, which emphasizes active collaboration with family members and inclusion of family members in the treatment of hospitalized persons with SMI, as opposed to the control and dominance of professionals (Dunst et al., 2007; Franck et al., 2013).

The FCC model was developed most notably for pediatric care (Kokorelias et al., 2019), and implementing it in the context of psychiatric care presents unique challenges. The biomedical model continues to influence traditional healthcare practice in psychiatric services, with a focus on the individual with SMI rather than the family as the recipient of care (Hsiao et al., 2019). Based on recognition of the significant role that family members may play in the treatment process of a hospitalized family member with SMI, and assuming that implementation of an FCC model could reduce the stressors and anxiety experienced by family members, a structured FCC model was developed and implemented in two psychiatric hospitals in Israel. A study was conducted to examine whether the implementation of the model makes a difference in the stress and anxiety experienced by caregivers of persons with SMI.

### The Stressors and Anxiety Experienced by Family Members During Psychiatric Hospitalization

Caregivers may experience high levels of distress due to factors such as the change in behavior and functioning of the family member with SMI that led to the psychiatric hospitalization, the experience of having a family member hospitalized in a psychiatric hospital, the restricted visitations in psychiatric hospitals, and the burden of playing the role of the caregiver (Ma et al., 2018). Particularly during the first hospitalization, stressors could also stem from a lack of experience in providing care for a person with SMI, limited knowledge regarding the early stages of the mental illness, and the need to acknowledge the reality of having a family member with SMI (Jones, 2009; Kumar et al., 2019; Lefley, 2009; Ma et al., 2018).

Giacco et al. (2017) reported that caregivers in inpatient mental health settings are often emotionally and physically exhausted, as well as frightened and worried for their relative or friend who was admitted to an inpatient psychiatric unit. In a qualitative study, Wilkinson and McAndrew (2008) found that their study participants experienced a sense of powerlessness once the person they were caring for was admitted to a psychiatric hospital. They described this period as a time of confusion and distress during which they needed support to understand and deal with what was happening.

A review of studies on the experience of caregivers of patients in psychiatric hospitals (Abou Seif et al., 2022) reveals that, despite the significant emotional toll taken on them by the hospitalization and the heightened need for support, their needs were seldom acknowledged. In this context, they strongly perceived inpatient staff as being unsupportive. One source of the stress that caregivers experience is the nature of their relationship with professionals during hospitalization. Jubb and Shanley (2002) addressed the failure of mental health professionals to recognize the potential therapeutic benefits of caregivers’ involvement in treatment programs in psychiatric hospitals, noting that in many cases, caregivers are not informed about the treatment being provided.

### The SAP Model

The principles of the FCC model served as a basis for developing the SAP model in Israel. The SAP – Support, Accompanying, and Partnership – model (Shalev, 2020; Shalev et al., 2017) was developed to meet the needs of caregivers and to address the challenges of involving them in the different stages of the psychiatric hospitalization of their family member with SMI. The caregiving role played by family members stems from their role in caring for the needs of the family member with SMI and providing him/her with support during the psychiatric hospitalization. The "support" component of the SAP model pertains to the caregivers’ need for support with their own difficulties during hospitalization. The "accompanying" component pertains to the need to accompany caregivers in finding ways to overcome barriers and difficulties they may encounter in their communication with the family member with SMI and in their attempt to provide him/her with support during the hospitalization (barriers that may stem from changes in the behavior of the family member with SMI due to a decline in their mental health). The "partnership" component is based on the underlying assumption that caregivers can be an important resource for the treatment of a family member with SMI. Mental health professionals in psychiatric wards may not be aware of the needs of family caregivers or lack the knowledge required to implement a family-centered model. Therefore, this model includes a protocol with clear guidelines for mental health professionals in psychiatric wards (psychiatrists, psychologists, social workers, and psychiatric nurses) for conducting three meetings with caregivers: one at the beginning of hospitalization, one at a more advanced stage of hospitalization, and one before the discharge of the family member with SMI from the hospital. The protocol specifies the overall goal and the specific objectives of each meeting as well. It also includes specific instructions for the mental health staff members regarding what to do in each meeting. The goal of the first meeting is for the mental health professionals and the caregivers to get to know each other and to develop a basis for mutual collaboration (e.g., providing information regarding a contact person in the psychiatric ward). The mental health professionals also offer an initial assessment of the family member’s situation and the kind of treatment being administered. In addition, based on the assumption that the caregivers possess valuable knowledge about the family member with SMI, the mental health professionals ask the caregivers to share with them information about the family member (e.g., the reasons for the hospitalization and past ways of coping with the mental illness). The goal of the second meeting is to expand the partnership with the family members. The mental health professionals share the treatment plan with the caregivers and provide them with opportunities to express their views and ask questions about the plan. They also explore the support needs of the caregivers during the hospitalization (e.g., by identifying the sources of stress they experience during the hospitalization and possible ways of mitigating it, and by accompanying them in fulfilling the caregiving role during the hospitalization). A study conducted with family members of persons with SMI who were hospitalized in psychiatric hospitals in Israel (Shor et al., 2022) indicates that family caregivers have difficulty acknowledging their need for help and seeking help for themselves due to factors such as the stigma associated with receiving help in psychiatric hospitals. Therefore, one objective of the mental health professionals during the second meeting is to help the family caregivers address their needs. The major goal of the third meeting is to develop, in collaboration with the family members, a discharge plan and a plan for rehabilitation in the community. Another is to prepare the family for the post-discharge period. The mental health professionals use a psychoeducational approach to provide the caregivers with information regarding how to handle the post-discharge period (e.g., how to best support the management of the illness and prevent relapse). The psychoeducational approach was one component of the intervention included in the SAP model, in addition to interventions aimed at developing a partnership with caregivers and providing them with support.

### Methods

The aim of the study was to examine whether the implementation of the SAP model had an impact on the stress and anxiety experienced by caregivers with a family member with SMI who is hospitalized in a psychiatric hospital. To this end, in two psychiatric hospitals in Israel, we conducted a comparative study between an intervention group, in which the SAP model was implemented, and a control group.

A comparative A/B method research design was implemented in four wards in two psychiatric hospitals in Israel, examining stress and anxiety in the participants. One hypothesis was that lower levels of stress and anxiety would be found in the intervention group, in comparison to the control group, in the second delivery of the questionnaire. Another was that, in the intervention group, lower levels of stress and anxiety would be found in the second delivery of the questionnaires, in comparison to the levels of stress and anxiety in this group in the first delivery of the questionnaires.

Institutional psychiatric care and treatment in Israel are provided by two types of facilities: specialized psychiatric hospitals and psychiatric inpatient units in general hospitals. Schizophrenia is the major diagnostic category of persons admitted to psychiatric institutions in Israel, and affective disorders are the second largest category (Aviram, 1996, 2010). The two psychiatric hospitals in which the study was conducted are specialized state-run psychiatric hospitals that operate according to the regulations of the Israeli Ministry of Health. The protocol-based SAP model was implemented in the intervention group, and the study used the A/B method of delivering the questionnaire at two different points in time. Questionnaires were delivered to caregivers of the hospitalized individual with SMI at the beginning of hospitalization and four weeks after discharge. This method was selected to examine changes in the stress and anxiety levels experienced by the family members between two points in time, as well as differences between the intervention and the control groups. The caregivers who were recruited for the study were family caregivers with significant personal relationships with hospitalized individuals with SMI and were involved in meeting their needs during the psychiatric hospitalization.

### Instruments

The questionnaire contained a background questionnaire about the caregivers and about the family member with SMI and his/her hospitalization. In addition, two instruments were utilized to assess the stressors and anxiety that the family caregivers experienced.

The Perceived Stress Scale is a widely used psychological instrument for measuring the perception of stress (Cohen et al., 1983), which was later revised and reduced into a 10-item scale, PSS-10 (Cohen & Williamson, 1988).^1^

This instrument assesses the degree to which situations in one’s life are appraised as stressful. It was developed to serve as a global, subjective measure of perceived stress. Items were designed to reflect how unpredictable, uncontrollable, and overloaded respondents find their lives. The scale also includes several direct queries about current levels of experienced stress. The questions on this scale ask about feelings and thoughts during the last month. Respondents are asked how often they felt a certain way. Each answer is scored on a 5-point Likert scale ranging from 0 (never) to 4 (very often), with higher scores indicating greater perceived stress. The scale includes six negative items assessing the degree of lack of control and negative reactions, as well as four positive items assessing the individual's ability to cope with existing stressors. There is some debate as to whether a 1 or a 2-factor model best describes the relationships among the PSS-10 items (Taylor, 2015). The 1-factor model was used in the study. In order to use the 1-factor model, the four positive items were reversed. The PSS has emerged as the most popular measure of perceived stress and has been validated on diverse samples (Taylor, 2015). In three national surveys conducted in the United States that utilized the PSS-10, Cohen and Janicki-Deverts (2012) found that stress was higher among women than men and increased as age, education, and income decreased. One of the surveys had a good internal consistency reliability (.79), and two enjoyed very good reliability (.91). In a study with family caregivers of persons with schizophrenia, Xiao et al. (2023) reported a good internal consistency reliability of.79 for the PSS, as measured by Cronbach’s alpha coefficient.

The Hospital Anxiety and Depression Scale (HADS) is a brief, self-administered rating scale (Zigmond & Snaith, 1983) consisting of 14 items (on a 4-point Likert scale, range 0–3): seven measuring anxiety (HADS-A) and seven measuring depression (HADS-D).^2^ According to Bjelland et al. (2002), Cronbach’s alpha coefficient of internal consistency was reported in 15 studies, ranging from medium (.68) to high (.93, mean =.82) for HADS-A and from medium (.67) to high (.90, mean =.82) for HADS-D. Evidence of the good concurrent validity of the HADS has been reported in psychiatric services, and good validity results were found in the implementation of this instrument in diverse clinical settings (Bramley et al., 1988).

### Procedure

Ethically, the study received the approval of the Helsinki Institutional Review Board of the two psychiatric hospitals in which the research was conducted. Acquiring participant consent to participate in the study occurred in two stages. A psychiatrist in each psychiatric ward made the initial contact with the family member with an SMI who was admitted to the psychiatric ward, explained the objectives of the study, and asked for their consent to contact a family member. If consent was given, the psychiatrist asked the family member with SMI to sign an informed-consent letter permitting the contact of a family member who functions as a caregiver. In the second stage, a mental health professional staff member established contact with the caregivers, explained the study, and asked them to sign an informed consent letter. Both the hospitalized persons and their caregivers were assured that refusal to participate in the study would not affect their eligibility for service. The study was presented to all the persons with SMI who were admitted to the closed wards included in the study and their caregivers, and 70 percent agreed to participate. Participant recruitment for each group lasted six months. Two research assistants arranged individual meetings with the caregivers and met with the caregivers in a room that allowed for privacy within the psychiatric hospital. The questionnaires were self-administered by the participants, except in situations in which caregivers had trouble understanding some of the wording in the questionnaire, as in the case of Muslim family caregivers, for whom Hebrew is not their first language.

The mental health staff members received 28 h of training to implement this model. One of the major aims of SAP training is to raise the awareness of the mental health staff members regarding family-centered work. Stanbridge and Burbach (2007) note that promoting the involvement of caregivers in mental health services is necessary for increasing awareness among professional staff members regarding the needs of caregivers, in order to create more caregiver-sensitive services. The training of the staff members included presentations and training regarding how to implement the protocol and how to conduct the three meetings. The mental health staff members in the intervention group followed the guidelines for conducting the three meetings. They implemented the three components of the model (Support, Accompanying, and Partnership) in each of the meetings but placed a different emphasis on each one, based on the changing needs of the caregivers during the different stages of the hospitalization. For example, there was most often a greater need for the accompanying component, supporting family members in their caregiving role, during the initial stage of the hospitalization. This, for example, included the provision of information regarding the mental health condition of the family member with SMI and the hospitalization process, and suggestions about ways the caregivers could support the family member with SMI. The mental health professionals in the control group did not rely on a protocol in the meetings and had no set number of meetings to be conducted with the family caregivers. They were in contact with the caregivers mainly to provide them with information about their family member with SMI or in response to an inquiry made by the caregiver. The frequency of the meetings varied in accordance with the needs of the caregivers and the nature of their relationships with the mental health professionals.

## Results

### Background of the Participants

Ninety-three caregivers participated in the first delivery of the questionnaire within the intervention group, and 63 caregivers participated in the second delivery. Seventy-five caregivers participated in the control group for the first delivery of the questionnaire, and 45 participated in the second delivery. Statistically significant differences were not found in a comparison of the background information of the participants, which utilized the Chi-square test of independence and the t-test for independent samples. Differences were not found in the age of the participants [intervention group: *M* = 48.98 (*SD* = 14.93); control group: *M* = 48.81 (*SD* = 12.07); *t*(166) =.080, *p* =.93], in the gender division [intervention group: 36.6% men, 63.4% women; control group: 34.7% men, 65.3% women; X^2^ (1, *N* = 168) =.065, *p* =.799], or in the identity of the caregivers [intervention group: 38.5% mothers, 12.1% fathers, 16.5% siblings, 25.3% spouses, and 6.6% children; control group: 46.7% mothers, 12% fathers, 24% siblings, 12% spouses, and 5.3% children; X^2^ (4, *N* = 168) =.6.516, *p* =.259]. Most of the caregivers in the intervention group (92.47%) and in the control group (92%) were Jewish, and their first language was Hebrew. The rest were Muslims or Christians, for whom Hebrew was a second language. However, all of them spoke Hebrew fluently. Differences were also not found in the age of the hospitalized family members with SMI [intervention group: *M* = 36.96 (*SD* = 14.36); control group: *M* = 36.2 (*SD* = 13.41); *t*(166) =.347, *p* =.729]. Most of the family members with SMI in both groups (intervention group: 85%; control group: 86.6%) were coping with schizophrenia, depression, or bipolar disorder. Most lived in the community with members of their families of origin or with a spouse when they were not hospitalized (intervention group: 72%; control group: 73.3%). For most of the participants in each group, this was their first hospitalization (68.8% in the intervention group and 65.3% in the control group). Statistically-significant differences were not found in the average number of days of psychiatric hospitalization during the hospitalization during which the research took place [intervention group: *M* = 29.34 (*SD* = 31.697); control group: *M* = 33.39 (*SD* = 60.062); *t*(166) =.599, *p* =.57].

### Comparison Between and Within Groups

The mean values of the sum of items in the first delivery of the questionnaires were as follows: for the PSS-10 scale, in the control group, *M* = 16.554 (*SD* = 7.820), and in the intervention group, *M* = 16.278 (*SD* = 7.889); for the HADS-A, in the control group, *M* = 7.621 (*SD* = 4.562), and in the intervention group, *M* = 6.491 (*SD* = 4.592); and for the HADS-D, in the control group, *M* = 9.00 (*SD* = 5.411), and in the intervention group, *M* = 8.327 (*SD* = 5.114). The mean values of the sum of items during the second delivery of the questionnaires were: for the PSS-10 scale, in the control group, *M* = 17.066 (*SD* = 9.062), and in the intervention group, *M* = 15.131 (*SD* = 7.902); for the HADS-A, in the control group, *M* = 7.129 (*SD* = 5.090), and in the intervention group, *M* = 4.737 (*SD* = 3.970); and for the HADS-D, in the control group, *M* = 8.103 (*SD* = 5.633), and in the intervention group, *M* = 8.229 (*SD* = 5.295).

A comparison of the means of the sums of items on the Hospital Anxiety and Depression Scale (HADS) and the Perceived Stress Scale (PSS-10) was conducted between the intervention and control groups utilizing the t-test for independent samples. Statistically significant differences were not found in a comparison of the means of the sum of the items of these scales at the first delivery of the questionnaires at the beginning of the psychiatric hospitalization. Statistically significant differences were also not found in the comparison of the PSS-10 scale at the second delivery of the questionnaire. However, a statistically significant difference was found in the comparison between the means of the sum of items of the anxiety subscale (HADS-A) in the second delivery of the questionnaire (t(106) = 2.478, *p* <.05), with a lower mean in the intervention group (*M* = 4.737, *SD* = 3.970) than in the control group (*M* = 7.129, *SD* = 5.090).

A comparison of the means of the sum of items in these scales was also conducted within each group between the first and second deliveries of the questionnaire. Statistically significant differences were not found in the comparison of the means of the PSS scale in the intervention group and the control group. Statistically significant difference was also not found in the within-group comparison of the HADS-A subscale in the control group. However, a statistically significant difference was found in a comparison of the mean of the HADS-A subscale in the intervention group [t(62) = 3.426, *p* =.001]. The mean of the sum of items of this subscale was statistically significantly higher in the first delivery of the questionnaire (*M* = 6.491, *SD* = 4.592) than in the second delivery of the questionnaire (*M* = 4.737, *SD* = 3.970).

Statistically significant correlations were not found between the mean of the participants’ age in each group and the mean of the items of each of the two scales and subscales in the control and intervention groups in both deliveries of the questionnaire.

## Discussion

The findings indicate a lack of statistically significant difference between the control and intervention groups in a comparison of the measures of stress (PSS) in the first and second deliveries of the questionnaires (the level of stress was medium-to-high in both measurements). The medium-to-high level of stress experienced by the participants in the first and second deliveries of the questionnaire could be explained by the fact that, for most of the participants in both groups, it was the first hospitalization of the family member with SMI, which was a period that could be characterized by a high level of stress for the caregivers. However, differences were found in the anxiety measure (of the HADS scale). The anxiety level was statistically significantly lower in the intervention group than in the control group in the second delivery of the questionnaire. In addition, in the within-group comparison of the intervention group, statistically significantly lower anxiety levels were found in the results of the second delivery of the questionnaires in comparison to the first delivery. These findings indicate that, despite the possible limitations of the SAP model in reducing the stress level of the caregivers, it could have a positive impact on the caregivers by reducing their anxiety level (from medium-to-high to medium).

The implementation of the SAP model in the intervention group could be a possible explanation for the differences in anxiety level. The three structured meetings between the mental health professionals and the family members, which were conducted according to the SAP model during the different stages of the psychiatric hospitalization, ensured the family members' access to mental health professionals and the exchange of information with the caregivers. The implementation of this model may have resulted in a lower anxiety level. Participation in the SAP meetings may have also facilitated an improvement in continued services after being discharged from the hospital, which may have also contributed to a lower anxiety level. Family members in Addington et al.’s study (2005) noted that without the support of the professional staff, they may experience difficulties coping with stressors and providing support for the hospitalized person. Family members in Tunnell et al.’s (1988) study also indicated that support, in the form of provision of greater knowledge and understanding with specific suggestions for coping with the behavior of the family member with SMI, was helpful in reducing anxiety.

Participation in the SAP meetings provided caregivers with opportunities to receive information at the initial stage of hospitalization, at a more advanced stage of hospitalization, and before discharge. In a study conducted with 86 hospitalized persons with SMI, their caregivers, and professional staff, Giacco et al. (2017) found that the provision of information was an important factor in reducing the stress level of the family members. Caregivers in their study reported instances in which a lack of information caused them to feel frustrated, worried, alone, or “in the dark.” The impact that a lack of information can have on stressors and anxiety experienced by caregivers of persons with SMI was also identified in other studies (Guarnaccia & Parra, 1996; Jubb & Shanley, 2002). Research indicates that caregivers value information about the mental illness and medications in question and wish to be provided with regular updates about service users’ progress and daily activities (Hsiao et al., 2019; Shankar & Muthuswamy, 2007).

## Implications

Research indicates that, instead of a time when caregivers receive a respite from caregiving, psychiatric hospitalization may be a time of increased stress that warrants recognition and acknowledgement by mental health professionals (Wilkinson & McAndrew, 2008). The findings of the present study illuminate the potential value of integrating the SAP model as an integral part of the work of professional staff in psychiatric hospitals, as its implementation can help reduce the level of anxiety experienced by family members. The implementation of the structured meetings of the SAP model during the different stages of psychiatric hospitalization could provide a context for communication between family members and mental health professionals in psychiatric hospitals. Research on family caregivers of persons with SMI hospitalized in psychiatric hospitals has found that family members value the opportunity for good communication with professionals, for example, about the treatment plan or about changes in the mental health situation of the hospitalized family member (Ewertzon et al., 2011). Opportunities to discuss these subjects with the mental health professionals and to receive updates about the family member with SMI can help reduce the anxiety experienced by family members.

The findings illuminate the significance of expanding the common model in psychiatric hospitals: the biomedical model, which focuses primarily on the individual with SMI. They highlight the need for a paradigm shift to a more collaborative and family-centered model in psychiatric hospitals. This could not only serve to reduce caregiver anxiety but also has the potential to enhance the family caregivers’ ability to support their family member with SMI during hospitalization and in the community. In order to create an environment in the psychiatric hospital that supports the implementation of a family-centered model, Wood et al. (2013) address the importance of having the hospital environment be experienced as "permeable" to caregivers. They recommend providing a therapeutic landscape that ensures not only the well-being of persons with SMI but also that of their family members. A permeable environment is one that enables caregivers to enjoy free movement across the interface between "inside" and "outside" the hospital setting and that fosters good relationships between the hospital staff and the caregivers. The restricted visitations that characterize the closed wards in psychiatric hospitals in Israel could be a barrier to creating a permeable environment. However, implementation of the SAP model could help overcome this barrier and create a more open and collaborative environment for caregivers in psychiatric hospitals. It could facilitate an overcoming of the barriers embedded in the very nature of psychiatric hospitalization as a result of restricted visitations, such as the limited information that family caregivers may possess about the hospitalized family member with SMI.

This comparative A/B study sheds light on the potential value of implementing the SAP model in reducing the anxiety level of family caregivers. There is, however, a need for further examination of which components of the model the caregivers experience as helpful in reducing the anxiety they felt during the psychiatric hospitalization of a family member with SMI. There is also a need to examine the experience of family caregivers from minority groups who may experience language and cultural barriers during implementation of the SAP model.

## Footnotes

1 PSS-10 contact information and permission to use: Mapi Research Trust, Lyon, France, https://eprovide.mapi-trust.org

2 This study was conducted based on an unauthorised Hebrew translation of the Hospital Anxiety and Depression Scales © (HADS). In no event shall the publisher GL Assessment nor Mapi Research Trust be liable for any direct, indirect, special, incidental or consequential damages arising out of any use of this translation.

## Declarations

### Permission to Reproduce Material

Permission was granted to reproduce material from another source (see note 1) and to include material with a disclaimer (see note 2).
